# Down-regulation of A-FABP predicts non-muscle invasive bladder cancer progression: investigation with a long term clinical follow-up

**DOI:** 10.1186/s12885-018-5137-4

**Published:** 2018-12-10

**Authors:** Christel Mathis, Isabelle Lascombe, Franck Monnien, Hugues Bittard, François Kleinclauss, Isabelle Bedgedjian, Sylvie Fauconnet, Séverine Valmary-Degano

**Affiliations:** 1 Department of Pathology, University Hospital of Besançon, F-25000 Besançon, France; 2 University Bourgogne Franche-Comté, EA3181, LabEx LipSTIC ANR-11-LABX-0021, F-25030 Besançon, France; 3 Department of Urology, University Hospital of Besançon, F-25000 Besançon, France

**Keywords:** Bladder cancer, A-FABP, FABP4, Adipocyte-fatty acid binding protein, Prognostic marker

## Abstract

**Background:**

Non-muscle invasive bladder cancers (NMIBC: pTa, pT1) are characterised by a high risk of recurrence and/or progression. Identification of prognostic markers is needed to improve both diagnosis and management of the disease. The aim of this study was to analyse the expression of A-FABP (adipocyte-fatty acid binding protein) and to evaluate its prognostic value in bladder cancer with a long term clinical follow-up.

**Methods:**

A-FABP expression was investigated by immunohistochemistry in 236 tumours (114 pTa, 61 pT1, 61 pT2–4). Immunostaining was classified as negative (absent or weak immunostaining and moderate or strong staining on ≤10% of cells) or positive (moderate or strong staining on > 10% of cells). Event-free survival (EFS) and overall survival (OS) were determined with a 87.3 months median follow-up in the overall cohort. Recurrence-free survival (RFS) and progression-free survival (PFS) were established in NMIBC**.**

**Results:**

Loss of A-FABP was associated with higher mean age, high stage/grade, and the presence of metastatic lymph nodes. It was correlated with shorter median EFS (17.5 vs 62.5 months; *p* = 0.001) and mean OS (76.7 vs 154.2 months; *p* = 0.009) and with higher risk of progression in the pTa/pT1 subgroup (HR, 0.36; 95% CI, 0.13–0.96; *p* = 0.041) and importantly in the pTa tumours (HR, 0.34; 95% CI, 0.10–0.97; *p* = 0.045).

**Conclusion:**

These results demonstrated that loss of A-FABP expression following a long follow-up was predictive of pTa and pTa/pT1 progression. Immunohistochemistry on diagnostic biopsy is easy to use and could be of value to help clinicians to propose appropriate treatment for these tumours.

## Background

Bladder cancer is a significant cause of morbidity and mortality worldwide with about 430,000 new cases diagnosed in 2012. In the European population, it represents 4% of all cancers with 151,297 new cases and 52,411 deaths in 2012 [1]. Over 90% of bladder cancers are urothelial carcinoma (UC) [2]. At diagnosis, 75% are non-muscle invasive bladder cancers (NMIBC). They include carcinoma in situ (C*is*) and papillary tumours confined to the mucosa (pTa) or lamina propria (pT1). The remaining 25% are muscle-invasive bladder cancers (MIBC, ≥pT2) [3]. In the NMIBC patient group, as many as 50 to 80% of cases with low grade pTa-pT1 will recur, and up to 40 to 50% of cases with high grade/G3 pTa-pT1 or associated C*is* will progress within 5 years to a higher tumour stage or metastatic disease [4]. The treatment of NMIBC involves transurethral resection associated or not with intravesical therapy [5]. Progression to (or at initial diagnosis presentation with) MIBC represents a critical step in disease progression. MIBC have a poorer prognosis, since 50% of patients will relapse with metastasis development within 2 years despite optimal therapy [6]. Standard therapy of organ-confined MIBC includes radical cystectomy or chemoradiotherapy [7]. Patients with bladder urothelial carcinoma should be carefully monitored for signs of disease recurrence or progression. The predictive ability of conventional clinical and pathological parameters is limited. To date, there are no established biomarkers that are able to forecast progression. Therefore, molecular prognostic markers of tumour recurrence and progression are urgently needed to improve our understanding, diagnosis and management of UC.

Several studies have reported the involvement of fatty acid binding proteins (FABPs) in the progression of different cancer types such as pancreas, breast, colorectal and renal cancers, and melanoma [8–13]. Nevertheless, the relationship between the expression pattern of the different FABPs in human cancer tissues and their role in cancer development is unclear. FABPs are lipid carriers involved in secretion, uptake and intracellular fatty acid transport to subcellular organelles such as mitochondria and peroxisomes [14]. They are implicated in glucose metabolism and lipid oxidation [15]. Adipocyte-FABP (A-FABP/FABP4/aP2) is an adipokine that binds hydrophobic ligands such as saturated or unsaturated long-chain fatty acids. It is highly expressed in adipocytes and macrophages, and has been linked to the development of insulin resistance, metabolic syndrome and atherosclerosis [16]. In addition, this protein works as a cytoplasmic shuttle protein for ligand activation of the nuclear receptor PPARγ to activate its downstream transcriptional targets involved in cellular differentiation, apoptosis and anti-inflammatory responses [17]. The exact function of this protein in cancer is still controversial. A-FABP implication has been explored in breast cancer [18, 19], ovarian cancer [20, 21], oral squamous cell cancer [22], and non-small cell lung cancer [23]. Few studies have investigated the expression of A-FABP in bladder cancer. This protein is highly expressed in normal urothelium [24, 25]. Proteomic analysis of protein expression profiles of bladder UC has highlighted a loss or decrease of A-FABP in high-grade/stage lesions compared with low-grade/stage tumours [24, 26]. The loss of A-FABP protein [25] or mRNA expression [27] has been associated with bladder cancer progression. However, in these studies, no data were available on patient follow-up.

The purposes of the present work were firstly, to study by immunohistochemistry the expression of A-FABP according to clinical and pathological parameters, and secondly, to evaluate its prognostic value in a cohort of 236 UC with a long follow-up. In particular, we investigated whether this protein could be a prognostic marker of recurrence or progression of pTa/pT1 UC.

## Methods

### Patient material

All primary UC diagnosed on transurethral resection of bladder tumours (TURB) in the Department of Pathology, Jean Minjoz University Hospital (Besançon, France) from 1 January 2000 through 31 December 2009 were eligible for inclusion in this retrospective study. We included all initial diagnoses of bladder UC from pTa to pT4 of any grade, before any treatment, a total of 274 patients. We excluded kidney, prostate or ureter cancers associated with bladder cancer. We also excluded patients without clinical information or usable samples (tissue bloc fully utilised and two negative internal controls). The medical records of all patients included were checked up to 15 May 2017 to determine follow-up. All living patients were informed of the study in writing and their consent was obtained. Tumour recurrence was defined as the reappearance of UC at a lower or equivalent pathological stage after completion of TURB. Tumour progression was characterised by a recurrence of disease with higher grade, stage or metastatic status.

### Cell lines and culture

The human cervical carcinoma cell lines HeLa, Ca Ski, C-33 A and the human bladder cancer cell line RT4 were obtained from ATCC (Rockville, MD, USA). Cells were maintained in DMEM (HeLa, Ca Ski), EMEM (C-33 A) or McCoy’s 5A medium (RT4) supplemented with 10% fetal calf serum (FCS) (Lonza, Basel, Switzerland), 1% antibiotic antimycotic mixture (100 μg/ml streptomycin, 100 U/ml penicillin, 25 μg/ml amphotericin B), 2 mM glutamine and 15 mM Hepes (Sigma-Aldrich, Saint Quentin Fallavier, France) at 37 °C in a humidified 5% CO_2_, 95% O_2_ air incubator.

### Protein extraction and western blotting analysis

Cells were washed with cold PBS 1X and scraped in RIPA lysis buffer (50 mM Tris-HCl pH 7.4, 0.1% SDS, 150 mM NaCl, 1 mM EDTA, 1% Nonidet P-40, 0.5% sodium desoxycholate) supplemented with protease inhibitors (Roche Diagnostics, Meylan, France). Whole cell lysates were then sonicated and centrifuged at 10000 rpm for 10 min at 4 °C. Protein concentration was estimated using the Bradford protein assay (Bio-Rad, Marnes-la-Coquette, France). Total protein extracts (30 μg) were dissolved in Laemmli buffer (Bio-Rad) and separated by 15% SDS-PAGE. Proteins were transferred onto PVDF membranes (GE Healthcare, Amersham, UK) and non-specific binding was blocked in TBS-Tween 20 buffer (0.5 mM Tris-HCl, 45 mM NaCl, 0.05% Tween 20, pH 7.4) containing 5% nonfat milk. Membranes were incubated with the primary antibody anti-A-FABP (clone AB13979, 1:1000, Abcam, Paris, France). Protein blots were probed with anti-β-actin (clone AC-15, 1:40000, Sigma-Aldrich) as controls for protein loading. Bound primary antibody was detected using an HRP-conjugated secondary antibody anti-rabbit IgG (1:5000 or 1:10000) obtained from BD Biosciences (Le Pont de Claix, France). Proteins were visualised using an enhanced chemiluminescence detection method (GE Healthcare) followed by film exposure (Hyperfilm ECL, GE Healthcare), or by using ChemiDoc XRS+ with Image Lab Software (Bio-Rad).

### Immunohistochemistry

Tissue samples, obtained after TURB, were fixed in 4% formalin and paraffin embedded. Blocks were cut serially at 3μm thickness, deparaffinised in toluene, and rehydrated in graded ethanol. Antigen retrieval was performed by using 0.5% H_2_O_2_ for 30 min, followed by unmasking in citrate buffer (pH 6.0) for 20 min at high temperature, and then blocked with 0.5% bovine serum albumin (BSA). Sections were incubated with the A-FABP primary antibody (rabbit anti-human FABP4; dilution 1:1500; Abcam ab13979) for one hour at room temperature using the automated IHC/ISH slide staining BenchMark XT instrument (Roche Diagnostics) according to the manufacturer’s instructions. After washing, the slides were incubated for 30 min with the ImmPRESS™ HRP Universal Antibody (anti-mouse IgG/anti-rabbit IgG, peroxidase) (Vector Laboratories, CliniSciences, Nanterre, France). Endogenous peroxidase activity was removed by dipping the sections in 5% hydrogen peroxide for 10 min at room temperature followed by incubation with streptavidin-horseradish peroxidase for 25 min. Finally, peroxidase activity was revealed by DAB (3,3′-diaminobenzidine) staining (0.9 mg/mL) for 7 min. Sections were counterstained with Harris haematoxylin/eosine/safran (HES) with Leica Autostainer XL (Leica Biosystems, Nanterre, France), dehydrated in alcohol, and mounted using a standard procedure. Negative controls were obtained by omitting the first antibody. Normal bladder specimens were obtained from patients who had undergone cystoprostatectomy for prostate carcinoma. For immunocytochemistry analysis, RT4 and Ca Ski cells were fixed with formalin, paraffin-embedded and processed as described above. The status of A-FABP was assessed in a coded manner by a pathologist without knowledge of the clinical or pathological features of the patient. For each section, the presence of A-FABP immunostaining in endothelial cells was checked as an internal control. The proportion of stained cells, the cellular localisation of immunostaining (nuclear, cytoplasmic, or both), the intensity and different types of staining (basal cells only; one-third, two-thirds or the entire height of the urothelium and/or patchy staining) were used as criteria of evaluation. A-FABP staining was considered positive when > 10% of cells were moderately or strongly stained, and negative when staining was weak or when ≤10% of cells were moderately or strongly stained.

### Statistical analysis

We used the mean (± standard deviation) values and frequencies (percentages) for the description of continuous and categorical variables, respectively. Means and proportions were compared using Student’s *t* test and the chi-squared test (or Fisher’s exact test, if appropriate), respectively.

Event-free survival (EFS) was calculated from the date of TURB to the date of the first recurrence, progression or death from any cause. If no event was observed, patients were censored at the last follow-up. Overall survival (OS) was defined as the time between the date of TURB and the date of last follow-up or the date of death from any cause. Recurrence-free survival (RFS) and progression-free survival (PFS) were defined as the time between the date of TURB and the date of the first tumour recurrence or tumour progression, respectively. Survival curves were built using the Kaplan-Meier method and compared using the log-rank test. Survivals were described as median with 95% confidence intervals (CI) for PFS, and OS or hazard ratio (HR) with 95% CI for RFS and PFS when medians were not reached. For EFS and OS, all variables with *p* <  0.05 observed in univariate survival analysis were included in a multivariate Cox regression model with stepwise backward elimination to estimate HR with a 95% CI and to select potential prognostic factors. Follow-up was calculated using reverse Kaplan-Meier estimation. All statistical tests were 2-sided and probability values < 0.05 were regarded as significant. Analyses were performed with SPSS 20 software (IBM).

## Results

### Patient characteristics

A consecutive series of 236 patients was investigated, including 192 men (81.4%) and 44 women (18.6%) with a sex ratio of 4.4. The mean age was 70.45 years (median 72, range 25–99) with a standard deviation of 13.3 years. Two pathologists reclassified all tumours from the old to the new 2004 WHO consensus classification to establish the following cohort: 114 pTa (25 papillary urothelial neoplasms of low malignant potential (PUNLMP), 69 low grade UC (LG-UC) and 20 high grade UC (HG-UC)), 61 pT1 (7 LG-UC and 54 HG-UC), and 61 pT2–4 (HG-UC). The subtype of 215 (91.1%) tumours was the common papillary histological type. The other subtypes were represented by UC with squamous, glandular, or neuroendocrine differentiation, as well as the micropapillary, reversed, and sarcomatoid variants. UC-associated carcinoma in situ was noted in 9/236 (3.8%) of pathological reports.

### Validation of a human anti-A-FABP specific antibody

**Fig. 1:**
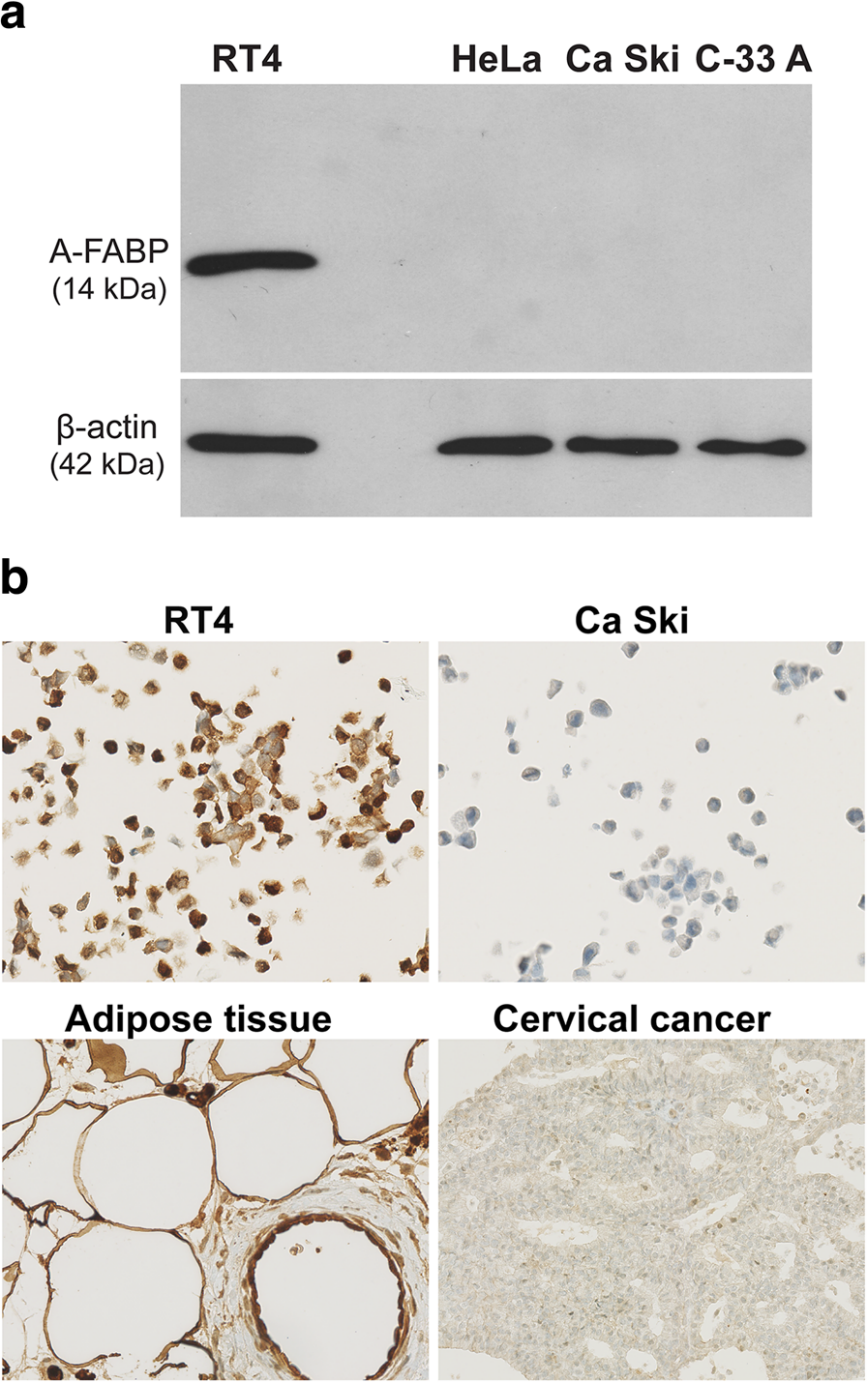
Positive and negative controls of A-FABP expression. **a** Western blots: bladder cancer RT4 cells were positive for A-FABP, while cervical cancer cells (HeLa, Ca Ski, C-33 A) were negative for A-FABP. B-actin was the protein loading control. **b** Immunohistochemistry: RT4 and adipose tissue were positive for A-FABP, while Ca Ski and cervical cancer (HPV+) were negative for A-FABP

Immunoblotting experiments were performed with total protein extracts from A-FABP-positive or -negative cancer cell lines. The Abcam human A-FABP antibody did not exhibit any aspecific hybridisation (Fig. 1a). It detected an A-FABP band of approximately 14 kDa size in the RT4 bladder cancer cell line (derived from a well-differentiated low grade papillary tumour) used as a positive control, as we have previously shown with a mouse A-FABP antibody [27]. On the other hand, the protein was not found in HeLa, Ca Ski and C-33 A cervical cancer cells. We showed by immunocytochemistry that A-FABP was present in RT4 cells and absent in Ca Ski cells, confirming the results obtained by western blotting. As expected, immunohistochemistry revealed that A-FABP was highly expressed in adipose tissue but was absent in cervical cancer specimens (Fig. 1b). These results validated the relevance of the choice of human A-FABP antibody.

### Histological analysis of human bladder biopsy samples and immunohistochemical evaluation of A-FABP expression

**Fig. 2:**
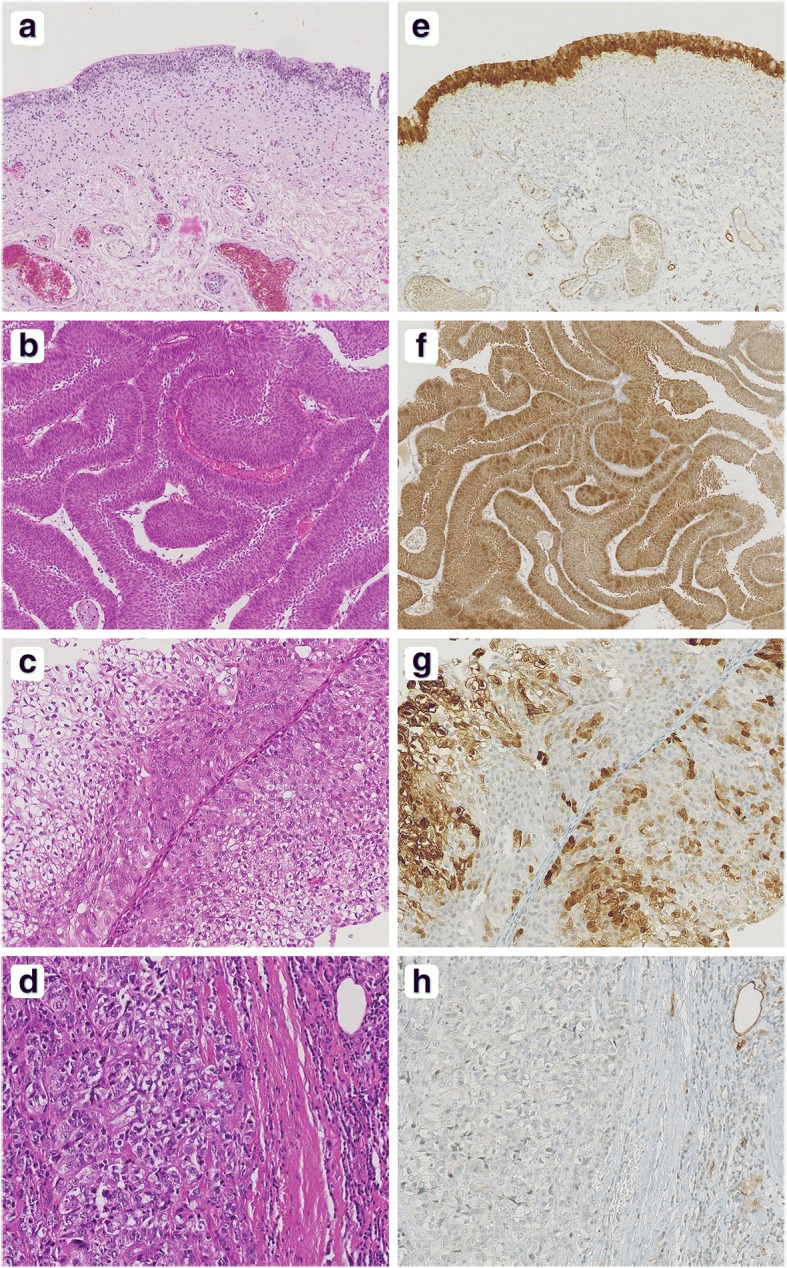
A-FABP expression in urothelial carcinoma. **a-d** Representative haematoxylin and eosin (H&E) staining. **e-h** A-FABP immunostaining. **a** Normal urothelium showing strong A-FABP staining in the entire height (**e**), magnification X10. **b** Low grade pTa papillary UC with strong A-FABP staining (**f**), magnification X10. **c** HG-UC pT1 with patchy A-FABP staining (**g**), magnification X20. **d** HG-UC pT3 invasive UC without A-FABP immunostaining (**h**), magnification X20

The expression pattern of A-FABP was analysed by immunohistochemistry on 236 tumour sections. The baseline characteristics of the patients are described in Table 1. Morphological analysis was performed on paraffin-embedded bladder biopsy tissue sections stained with haematoxylin and eosin (H&E). Healthy bladder tissue was used as positive control (Fig. 2a). The histology of the bladder tumours was clearly different from healthy bladder tissue. Papillary tumours are defined by the presence of true papillae with central fibrovascular cores covered by neoplastic epithelium. At low grade, there is a generally ordered architectural appearance to the cells within the epithelium with an impression of increased cellularity and increased nuclear density (Fig. 2b). The nuclei tend to be uniformly enlarged and retain the elongated to oval shape of normal urothelial cells, and the chromatin remains fine with small and generally inconspicuous nucleoli. At high grade, the papillae are frequently fused, forming apparently solid masses. The overall impression is one of disordered growth. The epithelium is of variable thickness. Individual and discohesive cells are haphazardly arranged within the epithelium. Nuclei are hyperchromatic and pleomorphic. The chromatin is dense, irregularly distributed and often clumped, while the nucleoli may be single or multiple and are often prominent. These characteristics are illustrated in Fig. 2c and d, corresponding to papillae from pT1 and an infiltrating contingent from pT3 UC, respectively. By immunohistochemistry, intense nuclear and/or cytoplasmic A-FABP staining was observed in the entire height of the normal urothelium (Fig. 2e). Of 236 UC specimens examined in this study, 102 (43.2%) were positive for A-FABP and 134 (56.8%) were negative. Of the 134 specimens considered as negative, 69 (51.5%) were totally negative, while 65 specimens (48.5%) displayed moderate or strong but patchy positive staining of less than 10% of tumour cells. Figure 2 illustrates different examples of immunostaining: a pTa UC with intense staining of more than 10% of tumour cells (Fig. 2f), a pT1 UC with heterogeneous staining of more than 10% of tumour cells (Fig. 2g), and a pT2 UC that was negative for A-FABP (Fig. 2h). Concerning the immunostaining pattern, A-FABP staining was strong and both nuclear and cytoplasmic in most cases (85.3 and 83.8%, respectively). We also studied the heterogeneity of the immunostaining as follows: absent, patchy, basal cell layer only, one-third, two-thirds, or the entire height of the urothelium. A-FABP positive tumours could simultaneously express different types of staining in the studied area. But in most cases, the entire height of the urothelium (68.6% UC) was involved and staining was patchy (62.7% UC).

**Table 1:** Clinicopathological data according to A-FABP expression

	N(%)	A-FABP		*P*
		Negative	Positive	
Population	236 (100)	134 (56.8)	102 (43.2)	–
Sex				
Male	192 (81.4)	111 (82.8)	81 (79.4)	0.503
Female	44 (18.6)	23 (17.2)	21 (20.6)	
Age				
Mean (sd)	70.45 (±13.3)	72.1 (±12.8)	68.3 (±13.8)	0.028
Histology				
Papillary	215 (91.1)	118 (88.1)	97 (95.1)	0.06
Variants*	21 (8.9)	16 (11.9)	5 (4.9)	
pT				
pTa	114 (48.4)	43 (32.1)	71 (69.6)	< 0.001**
pT1	61 (25.8)	39 (29.1)	22 (21.6)	
pT2	45 (19.1)	40 (29.9)	5 (4.9)	
pT3	5 (2.1)	4 (3.0)	1 (1.0)	
pT4	11 (4.7)	8 (6.0)	3 (2.9)	
pN				
0	206 (87.3)	110 (82.1)	96 (94.1)	0.006
1–2	30 (12.7)	24 (17.9)	6 (5.9)	
pM				
0	231 (97.9)	130 (97)	101 (99)	0.393
1	5 (2.1)	4 (3)	1 (1)	
Grade				
PUNLMP	25 (10.6)	6 (4.5)	19 (18.6)	< 0.001
LG-UC	76 (32.2)	30 (22.4)	46 (45.1)	
HG-UC	135 (57.2)	98 (73.1)	37 (36.3)	
C*is*				
yes	9 (3.8)	7 (5.2)	2 (2)	0.306
no	227 (96.2)	127 (94.8)	100 (98)	

*The histological variants include micropapillary, nested, reversed and sarcomatoid variants, urothelial carcinoma with squamous, glandular or neuroendocrine differentiation

**Due to some reduced numbers, the pT3 and pT4 stages were grouped together with the pT2 stage for the Khi-2 test

Abbreviations: *PUNLMP* Papillary urothelial neoplasm of low malignant potential, *LG-UC* Low grade urothelial carcinoma, *HG-UC* High grade urothelial carcinoma, *Cis* Carcinoma in situ

### Association between A-FABP expression and clinicopathological data

Potential associations between A-FABP expression and clinicopathological features were investigated. As shown in Table 1, among A-FABP-positive tumours, strong immunoreactivity was detected in pTa specimens. Indeed, of 102 A-FABP-positive cases, 71 (69.6%) were pTa, 22 (21.6%) were pT1, 5 (4.9%) were pT2, 1 was pT3 (1%) and 3 (2.9%) were pT4. Statistical analysis revealed a significant association between loss of A-FABP expression and tumour stage (*p* < 0.001). Concerning histological grade, in the A-FABP-positive subgroup, 19 (18.6%) were PUNLMP, 46 (45.1%) were LG-UC, and 37 (36.3%) were HG-UC. In the A-FABP-negative subgroup, 6 (4.5%) were PUNLMP, 30 (22.4%) were LG-UC and 98 (73.1%) were HG-UC. Negative A-FABP immunoreactivity was thus significantly correlated with high grade (*p* <  0.001). The presence of metastatic lymphatic nodes was also correlated with loss of A-FABP expression (*p* = 0.006). In addition, patients whose tumours still expressed A-FABP were on average younger than those with an A-FABP negative tumour: 68.3 vs 72.1 years old (*p* = 0.028). On the other hand, there was no association between A-FABP expression and sex, the presence of visceral metastases or associated C*is*. To conclude, A-FABP immunoexpression was significantly associated with the following clinicopathological parameters: age, stage, grade, and lymph node status. Loss of A-FABP expression was generally correlated with high histologic grade and high stage.

### Association of clinicopathological data and A-FABP expression with EFS and OS

**Fig. 3:**
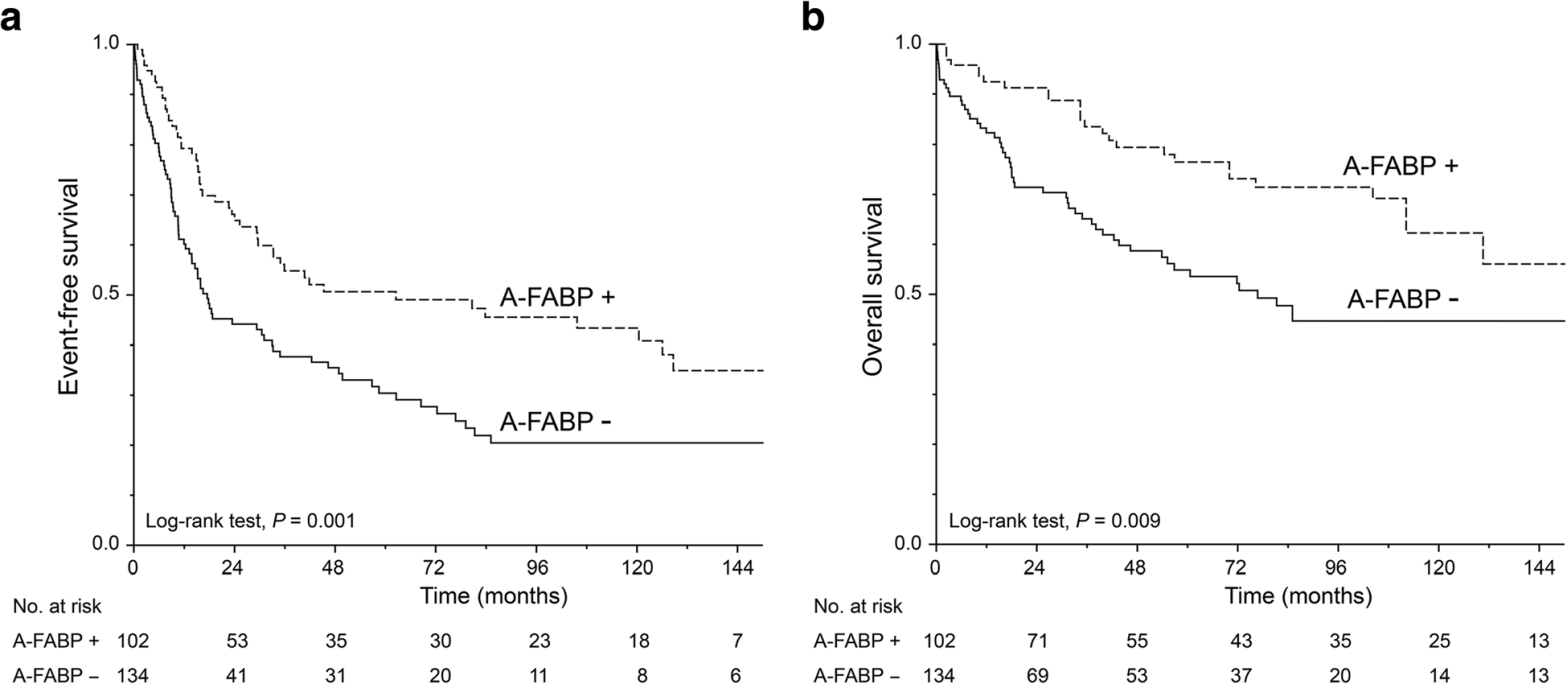
Kaplan-Meier plots for negative and positive A-FABP expression in the overall cohort. **a** Event-free survival, **b** Overall survival

An univariate statistical analysis was carried out in patients with UC to correlate EFS and OS with different clinical and histological parameters and with A-FABP expression (Table 2, Fig. 3a and b). During the period of the study, 126 (53.4%) patients presented an event (recurrence, progression, or death) and 110 (46.6%) did not. Younger patients (≤ 72 years old) had a longer median EFS (mEFS) than older patients (41.9 months vs 17.5 months; *p* = 0.005). A longer mEFS was also associated with pTa stage (72.3 months vs 14.8 for pT1 and 15.9 for pT2–4; *p* <  0.001), the absence of metastatic lymph nodes (34.9 months vs 15.2 months; *p* = 0.005) as well as with low grade (mEFS not reached for PUNLMP, 35.9 months for LG-UC and 17.5 months for HG-UC; *p* = 0.002). The univariate analysis showed that patients with a papillary tumour had a longer median OS (mOS) (149.3 months vs 33.2 months for patients with a histological variant; *p* = 0.032). Shorter mOS was associated with high stage pT2–4 (18 months vs 81.3 months for pT1, not reached for pTa; *p* < 0.001), the presence of metastatic lymph nodes (15.2 months vs 154.2 months; *p* < 0.001), the presence of metastases (16.5 months vs 149.3 months; *p* < 0.001), and high grade (not reached for PUNLMP, 178.5 months for LG-UC and 56.3 months for HG-UC; *p* < 0.001). Importantly, patients whose UC still expressed A-FABP had longer EFS (62.5 vs 17.5 months, respectively; *p* = 0.001) as well as longer OS (154.2 vs 76.7 months; *p* = 0.009) than those whose UC were A-FABP negative. A-FABP positive expression is an indicator of a good prognosis for the clinical outcome of patients with NMIBC (pTa/pT1). It could be a suitable tool for clinicians to distinguish patients with NMIBC who will progress from those who will not.

**Table 2:** Univariate and multivariate analyses of clinicopathological data and A-FABP expression in relation to EFS and OS of patients with urothelial carcinoma

	Univariate analysis				Multivariate analysis				Univariate analysis				Multivariate analysis			
	mEFS	95% CI		*p*	HR	95% CI		*p*	mOS	95% CI		*p*	HR	95% CI		*p*
		–	+			–	+			–	+			–	+	
Sex																
Male	29.3	17.1	41.5	0.371					128.4	91.9	165.0	0.696				
Female	33.2	0.0	88.0						not reached							
Median Age																
≤ 72 years	41.9	6.3	77.4	0.005	1.6	1.1	2.2	0.01	not reached			0.002	2.0	1.3	3.2	0.002
> 72 years	17.5	4.5	30.4						72.3	48.0	96.6					
Histology																
Papillary	30.5	14.2	46.9	0.702					149.3	108.1	190.5	0.032				
Variants	29.6	27.1	32.2						33.2	28.1	38.2					
pT																
pTa	72.3	34.8	109.8	< 0.001	1.4	1.1	1.7	0.003	not reached			< 0.001	2.1	1.6	2.9	< 0.001
pT1	14.8	7.7	22.0						81.3	47.1	115.5					
pT2–4	15.9	11.2	20.6						18.0	7.1	29.0					
pN																
0	34.9	16.5	53.2	0.005					154.2	107.8	200.5	< 0.001	2.1	1.1	4.0	0.028
1–2	15.2	6.7	23.7						15.2	6.7	23.7					
pM																
0	33.2	20.4	45.9	0.059					149.3	110.4	188.3	< 0.001				
1	16.5	3.7	29.3						16.5	3.7	29.3					
Grade																
PUNLMP	not reached			0.002					not reached			< 0.001				
LG-UC	35.9	7.9	63.9						178.5	122.5	234.6					
HG-UC	17.5	10.2	24.8						56.3	25.6	87.1					
C*is*																
no	30.5	19.1	42.0	0.258					128.7	89.4	167.9	0.789				
yes	81.3	0	181.4						81.3	0	166.4					
A-FABP																
negative	17.5	9.9	25.0	0.001					76.7	50.0	103.5	0.009				
positive	62.5	1.8	123.2						154.2	114.9	193.4					

Abbreviations: *EFS* Event-free survival, *OS* Overall survival, *PUNLMP* Papillary urothelial neoplasm of low malignant potential, *LG-UC* Low grade urothelial carcinoma, *HG-UC* High grade urothelial carcinoma, *Cis* In situ carcinoma

We performed a multivariate analysis including significant parameters of the univariate analysis (age, stage, lymph node status, grade and A-FABP expression). The multivariate Cox model applied to the resulting parameters showed that age (≥ 72 years) and stage could be considered as independent prognostic factors for EFS (*p* = 0.01 and *p* = 0.003, respectively) as well as age, stage and lymph node status for OS (*p* = 0.002, *p* < 0.001 and *p* = 0.028, respectively). A-FABP expression was not recognised as an independent significant prognostic marker.

### Association of clinicopathological data and A-FABP expression with RFS and PFS in pTa and pT1 tumours

**Fig. 4:**
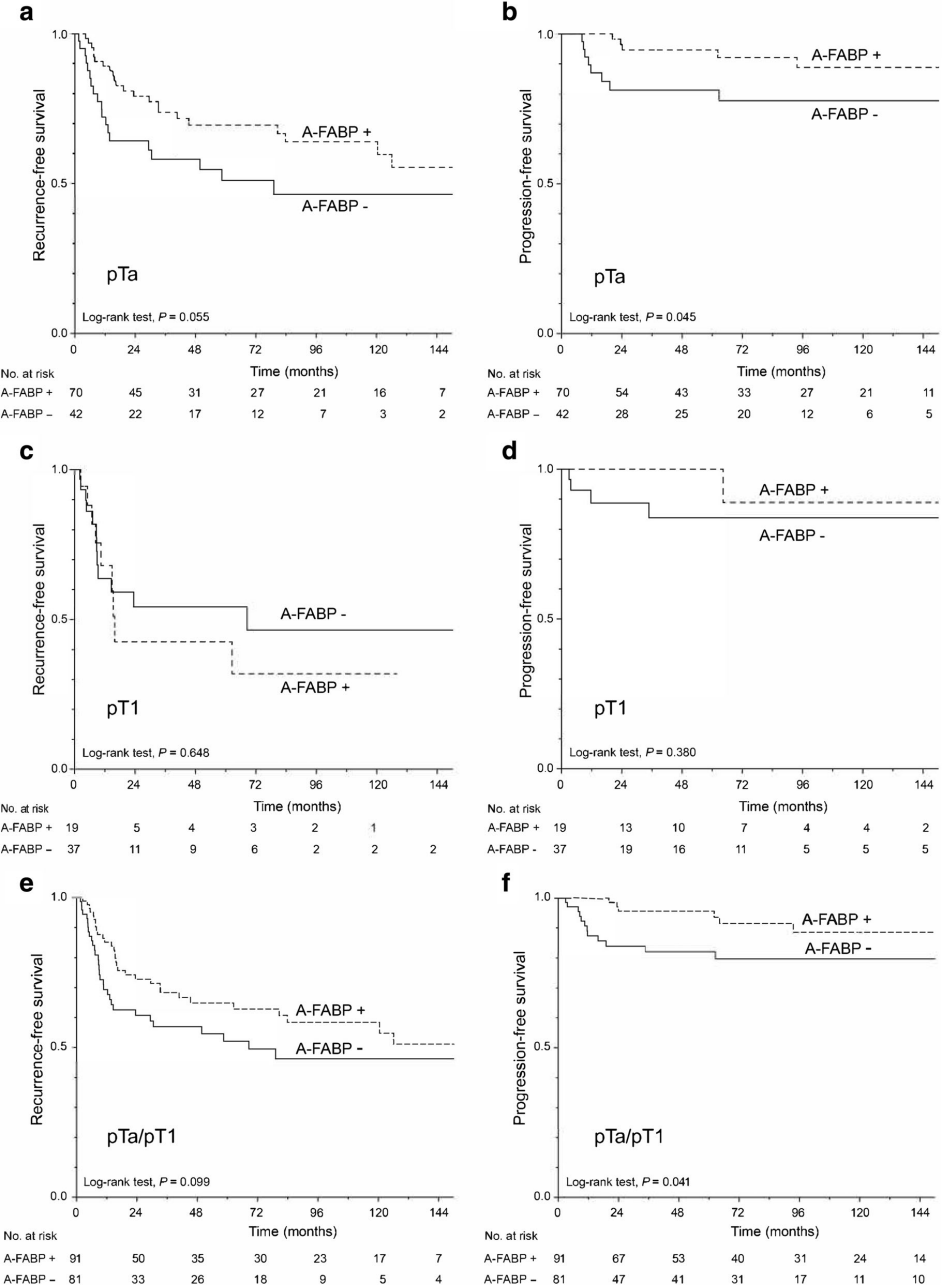
Kaplan-Meier plots for negative and positive A-FABP expression in pTa (**a, b**), pT1 (**c, d**) and pTa/pT1 (**e, f**) patients. **a, c, e** Recurrence-free survival; **b, d, f** Progression-free survival

Table 3 gives the results of RFS and PFS using univariate Cox analysis. Kaplan-Meier curves were plotted according to A-FABP expression (Fig. 4) in the pTa, pT1 and pTa/pT1 UC. Sex, age, histology of the tumour, grade, and C*is* were not associated with RFS or PFS. On the other hand, pT1 tumours had a higher risk of recurrence than pTa (HR, 1.71; 95% CI, 1.01–2.90; *p* = 0.048). Patients treated for pTa tumours with negative expression of A-FABP had a higher risk of progression (HR, 0.34; 95% CI, 0.10–0.97; *p* = 0.045). The risk of progression was not significant in pT1 UC patients with a negative expression of A-FABP. Patients in pTa/pT1 group with negative expression of A-FABP had a higher risk of progression (HR, 0.36; 95% CI, 0.13–0.96; *p* = 0.041).

**Table 3:** Univariate analysis of clinicopathological data and A-FABP expression in relation to RFS and PFS of patients with NMIBC

	HR	RFS		*P*	HR	PFS		*P*
		95% CI				95% CI		
		–	+			–	+	
pTa								
Sex (F)	0.60	0.24	1.53	0.280	0.04	0.00	22.1	0.111
Age (> 72)	1.21	0.65	2.28	0.547	1.16	0.38	3.54	0.800
Histology (variants)	0.99	0.14	7.26	0.997	0.05	0.00	404,941.26	0.574
Grade (for each change of category)	1.14	0.71	1.83	0.564	1.27	0.54	2.99	0.763
*Cis* (yes)				NA				NA
A-FABP negativity	0.55	0.3	1.02	0.055	0.34	0.10	0.97	0.045
pT1								
Sex (F)	0.04	0.00	16.09	0.102	4.34	0.41	46.4	0.187
Age (> 72)	2.04	0.84	5.00	0.110	0.75	0.12	4.6	0.756
Histology (variants)	0.04	0.00	129.34	0.245	0.05	0.00	18,539,252.39	0.646
Grade (for each change of category)	0.58	0.17	1.96	0.373	25.34	0.00	2,740,610.19	0.392
*Cis* (yes)	0.03	0.00	3.65	0.151	0.04	0.00	1840.31	0.350
A-FABP negativity	1.22	0.51	2.91	0.648	0.39	0.04	3.48	0.380
pTa/pT1								
Sex (F)	0.43	0.17	1.09	0.074	0.38	0.05	2.86	0.347
Age (> 72)	1.52	0.92	2.51	0.104	1.01	0.39	2.63	0.976
Histology (variants)	0.48	0.07	3.47	0.468	0.05	0.00	11,861.4	0.631
Stage (pT1)	1.71	1.01	2.9	0.048	1.06	0.38	2.99	0.907
Grade (for each change of category)	1.28	0.89	1.83	0.183	1.27	0.65	2.48	0.487
*Cis* (yes)	0.05	0.00	5.19	0.201	1.3	0.17	9.79	0.797
A-FABP negativity	0.66	0.40	1.08	0.099	0.36	0.13	0.96	0.041

*Abbreviations: *NMIBC (pTa and pT1)* Non-muscle invasive bladder cancer, *CI* Confidence interval, *HR* Hazard ratio, *RFS* Recurrence-free survival, *PFS* Progression-free survival

## Discussion

Non-muscle invasive bladder cancers are a heterogeneous group of urothelial tumours displaying the complexity of molecular alterations during bladder carcinogenesis. Significant progression risk factors currently highlighted are the presence of C*is*, pT1G3 tumours, multifocal carcinoma and lymph node status [28, 29]. Nevertheless, these histopathological parameters cannot forecast the long-term outcome of bladder cancer. It is therefore urgent to develop and validate in clinical practice useful prognostic and predictive biomarkers in order to identify patients at high risk of progression.

In the present work, we focused our attention on the A-FABP protein. We evaluated by immunohistochemistry the association of A-FABP status with clinicopathological features and prognostic outcomes in patients with UC. We confirmed the high expression of this protein in healthy urothelium, as previously described [24, 25]. We demonstrated that loss of A-FABP expression was correlated with more advanced age, the presence of metastatic lymph nodes, and, most importantly, with high tumour stage and histological grade. Another study examined A-FABP expression by immunohistochemistry [25]. The authors classified the immunostaining into six types according to its intensity and distribution. However, in clinical practice, these types are not easily applicable. For this reason, we proposed assessing positivity according to threshold and intensity of A-FABP staining. We considered as positive a moderate or strong staining of more than 10% of tumour cells, which was easy to use in routine. In our study, A-FABP staining in most cases was both nuclear and cytoplasmic, and in the majority immunostaining was strong. We also studied the heterogeneity of immunostaining as follows: absent, patchy, basal cell layer only, one-third, two-thirds, or the entire height of the urothelium. A-FABP positive tumours could simultaneously express different types of staining in the studied area. However, in most cases, the entire height of the urothelium was involved and showed patchy staining (data not shown).

In our work, survival analyses (EFS and OS) showed that the prognosis of bladder cancer depended on age, stage, grade and metastatic lymph node status. While the presence of C*is* is recognised to be a factor of poor prognosis, we failed to confirm this. This is probably due to the low number of C*is* in our cohort, perhaps because TURB essentially involved papillary tumours. In addition, the univariate survival analysis showed that A-FABP positivity was associated with a better prognosis for EFS and OS. However, we failed to identify A-FABP as an independent factor in the multivariate analysis. Finally, our data demonstrated that the presence of A-FABP was predictive of the absence of any event (recurrence, progression or death) and that loss of A-FABP expression in resected primary pTa UC, and pTa/pT1 group was a higher risk factor of progression. The decrease of A-FABP protein level may be used to precisely identify subsets of patients with NMIBC that have a poorer prognosis.

A-FABP has attracted increasing interest in recent years. Several studies have identified high serum levels of this protein as a useful prognostic marker for metabolic disorders, such as obesity, metabolic syndrome, type 2 diabetes and atherosclerosis [30–33]. While A-FABP was initially described in adipocytes and macrophages, the expression of this protein has currently been demonstrated in different cell types and in particular in some tumours. Nevertheless, the role of A-FABP in cancer is controversial. It could act either as a tumour suppressor or as an oncogene depending on tumour type. Thus, contrary to what we have demonstrated in bladder cancer, where strong expression of A-FABP was associated with a good prognosis, high A-FABP expression was significantly associated with shorter disease-free survival and OS in breast cancer patients [18]. In NSCLC, it was correlated with higher TNM stage and associated with shorter overall survival, and was an independent poor prognostic factor [23]. Up-regulation of A-FABP expression has been reported in metastatic human ovarian cancer compared with primary ovarian tumours [20]. An immunohistochemical study carried out in squamous cell carcinomas showed, in the same tissue sample, significantly higher expression of A-FABP in the tumour area of tongue SCC than in the non-tumour area. In addition, the protein knock-down with a specific siRNA prevented the proliferation of several SCC cell lines [22]. Overexpression of FABP4 has also been reported in glioblastoma [34].

On the other hand, other studies showed similar results to those we observed in UC. Lower expression of A-FABP was observed in human prostate cancer compared with normal prostate epithelial cells [35]. In the same way, A-FABP was detected in normal liver cells but not in hepatoma cells [36]. Different studies have examined mRNA and protein expression profiles in normal bladder urothelium and in UC with various histopathological grades and stages using diverse technical approaches (two-dimensional polyacrylamide gel electrophoresis, microsequencing, mass spectrometry, two-dimensional gel protein database approach for polypeptide identification, tissue microarray, immunohistochemistry, RT-PCR). All of these investigations revealed downregulation of A-FABP in invasive UC [24, 25], and good association of loss of A-FABP with tumour stage and grade [25]. In a previous study, we also reported that the decrease of *a-fabp* transcript level was significantly associated with high tumour stage and histological grade [27]. In addition, 2D-PAGE and RT-PCR analyses showed that protein abundancy was correlated with *a-fabp* mRNA levels, indicating that A-FABP expression was regulated transcriptionally or post-transcriptionally rather than at the translational level [37]. EFS, OS, RFS as well as PFS were not analysed in these studies.

Unlike the studies described above reporting the expression of A-FABP protein by immunohistochemical or two-dimensional electrophoresis analyses, we show on a long-term follow-up of patients that high expression of A-FABP was associated with a good prognosis and that the decrease of A-FABP expression is a tumor progression marker of pTa and pTa/pT1 group. Our study has attributed a prognostic value to the diminution of A-FABP. Another argument indicating that the absence of A-FABP could favour tumour progression is the elevated expression of the *a-fabp* gene observed in a non-C*is* group of tumours compared with a group with adjacent C*is* [38]. The mechanisms underlying the loss of A-FABP expression in UC have yet to be elucidated. Several hypotheses can be put forward based on the data in the literature, such as a polymorphism or epigenetic modifications. In triple-negative breast cancer, a single nucleotide polymorphism (SNP) of the 3’-UTR region of *a-fabp* gene has been shown to be associated with significantly lower expression of the protein [39]. The authors suggest that this SNP could distinguish the patients with high risk of recurrence. The alteration of DNA methylation patterns has been linked to carcinogenesis. In particular, hypermethylation was associated with tumour suppressor gene silencing [40]. This epigenetic modification is under the control of the DNA methyltransferases (DNMT). Thus, hypermethylation of the CpG islands around the human FABP4 promoter could be involved in the loss of FABP4 expression. Increased DNMT1 expression has been reported in human transitional cell carcinoma of the bladder. Interestingly, protein expression was higher in flat carcinoma in situ. Increased DNMT1 expression was also significantly correlated with histological grade [41].

It should be noted that the results of the present study are in contradiction with those of Wild et al. which found that high FABP4 expression was associated with pTa UC progression [42]. These authors demonstrated that FABP4 expression was up-regulated in 17 of 21 pTa samples with progression and down-regulated in 21 of 24 pTa tumours without progression. However, this study was carried out on mRNA by a combination of laser microdissection and gene expression profiling, and it was not validated by immunohistochemistry.

## Conclusions

In our pathological practice, A-FABP could be a helpful prognostic marker for bladder UC, easy to detect on formalin-fixed paraffin embedded tumour sections. Importantly, we demonstrated that loss of A-FABP expression was predictive of a higher risk of progression in pTa UC. This risk was not significant in pT1 and significant in the pTa/pT1 UC group. Bladder cancer is one of the most expensive to manage due to active surveillance following treatment of NMIBC. The identification of progression predictors is of great importance for the clinician in order to propose appropriate treatment and to improve the management of clinical follow-up of patients. Further prospective studies should be started to establish whether closer follow-up might be beneficial for patients with pT1 UC expressing low level or no A-FABP. Such follow-up could identify as early as possible patients at risk of progression. The surveillance strategy should be strengthened and more aggressive adjuvant therapy should be performed after initial curative surgery to achieve better prognosis. Immunohistochemical analysis of biomarkers on diagnostic biopsies is used in hospital routine for prognosis. In future perspectives, it could be interesting to study the prognostic value of this new biomarker on circulating A-FABP and/or urine A-FABP testing. This could be an easier way to follow the patients. In vitro functional studies are needed to elucidate the role of A-FABP in bladder cancer carcinogenesis. Highlighting the different signalling pathways controlling A-FABP expression is crucial to impact these regulatory networks involved in the disease. Given the results we have shown, we could postulate that maintaining a high level of A-FABP could prevent tumour progression. This protein is a target of PPAR and is induced by PPAR activators, as we have previously reported [27]. A pharmacological strategy using intravesical instillations of PPAR agonists could be applied to induce FABP4 expression in order to prevent tumour progression.

## Abbreviations

2D-PAGE: two dimensional-polyacrylamide gel electrophoresis
A-FABP: Adipocyte-fatty acid binding proein
BSA: Bovine serum albumin
CI: Confidence interval
Cis: Carcinoma in situ
DAB: 3–3′-diaminobenzidine
DNMT: DNA methyltransferase
EDTA: Ethylenediaminetetraacetic acid
EFS: Event-free survival
HES: Harris haematoxylin eosine safran
HG-UC: High-grade-urothelial carcinoma
HR: Hazard ratio
HRP: Horse radish peroxidase
IHC: Immunohistochemistry
ISH: In situ hybridization
LG-UC: Low grade-urothelial carcinoma
MIBC: Muscle invasive bladder cancer
NMIBC: Non-muscle invasive bladder cancer
NSCLC: Non small cell lung carcinoma
OS: Overall survival
PBS: Phosphate buffered saline
PFS: Progression-free survival
PPARγ: Peroxisome proliferator-activated receptor γ
PUNLMP: Papillary urothelial neoplasm of low malignant potential
RFS: Recurrence-free survival
RIPA: Radioimmunoprecipitation assay
RT-PCR: Reverse transcription-polymerase chain reaction
SCC: Squamous cell carcinoma
SDS-PAGE: Sodium dodecylsulfate-polyacrylamide gel electrophoresis
SNP: Single nucleotide polymorphism
TBS: Tris-buffered saline
TNM: Tumour node matastasis
TURB: Transurethral resection of bladder tumour
UC: Urothelial carcinoma
WHO: World Health Organization
